# Strategies for Articular Cartilage Repair and Regeneration

**DOI:** 10.3389/fbioe.2021.770655

**Published:** 2021-12-17

**Authors:** Yanxi Liu, Karan M. Shah, Jian Luo

**Affiliations:** ^1^ Shanghai Key Laboratory of Regulatory Biology, Institute of Biomedical Sciences and School of Life Sciences, East China Normal University, Shanghai, China; ^2^ Department of Oncology and Metabolism, The Medical School, The University of Sheffield, Sheffield, United Kingdom; ^3^ Shanghai Yangzhi Rehabilitation Hospital (Shanghai Sunshine Rehabilitation Centre), Tongji University School of Medicine, Shanghai, China

**Keywords:** articular cartilage, chondrocyte, microenvironment, regenerative medicine, articular cartilage repair, osteoarthritis

## Abstract

Articular cartilage is an avascular tissue, with limited ability to repair and self-renew. Defects in articular cartilage can induce debilitating degenerative joint diseases such as osteoarthritis. Currently, clinical treatments have limited ability to repair, for they often result in the formation of mechanically inferior cartilage. In this review, we discuss the factors that affect cartilage homeostasis and function, and describe the emerging regenerative approaches that are informing the future treatment options.

## Introduction

Articular cartilage (AC) is a highly specialized connective tissue that covers the surfaces of bones in a synovial joint. Its main function is to allow for an almost friction-less movement of the joints, and facilitate the transmission of loads whilst protecting the subchondral bone. However, the constant mechanical stresses that are experienced by the AC makes it vulnerable to wear, tears, and sports injuries. Moreover, mature AC has a limited ability to self-renew and repair as it is avascular, lacks innervation and has a low cell-density (Chiang and Jiang, 2009). Therefore, without sufficient and potent treatment, cartilage damage can easily lead to progressive tissue deterioration, debilitating joint pain, dysfunction, and eventually the degenerative disease, osteoarthritis (OA) (Borrelli et al., 2019).

Osteoarthritis refers to exposure of subchondral bone to the joint space, which causes inflammation that may be accompanied by osteophyte development, severe pain, and progressive loss of joint function. In fact, retrospective studies with arthroscopic examinations reveal the presence of chondral lesions in nearly 60% of the patients (Chubinskaya et al., 2015). Acute knee injury has also been shown to be a risk factor for OA, with individuals with cartilage damage 7.4 times more likely to develop the disease (Wilder et al., 2002). This is consistent with a more recent study in young adults which identified a risk difference of 8.1 for knee OA, between individuals with and without knee injury at 19 years of follow-up (Snoeker et al., 2020). With the improvement of human life expectancy and the development of social aging process, the incidence of OA will greatly increase during the following decades. In fact, epidemiological study in the Chinese population suggested that the prevalence of knee OA had reached 18% in the country (Wang et al., 2018).

Whilst current treatment options may improve joint function and reduce pain, they fail to elicit regeneration of the articular tissue with its distinct functional characteristics, and therefore are only partially effective. In this review, we begin by discussing the structure and biology of the AC, and then describe the current strategies for its repair, followed by highlighting the recent advances in regenerative technologies that are informing the future treatment options.

## Anatomy of the Articular Cartilage

*In utero* and at birth, AC is a compact, highly cellular tissue with isotropic distribution of chondrocytes, but poor in the extracellular matrix (ECM) (Weiss et al., 1968). As the AC matures and shapes before early adulthood, chondrocytes located in different areas receive AC variant degrees of mechanical, electrical, and physicochemical signals, leading to increased cellular size, accumulation of ECM, and eventually attaining a zonal anisotropic organization (Correa and Lietman, 2017).

**FIGURE 1:**
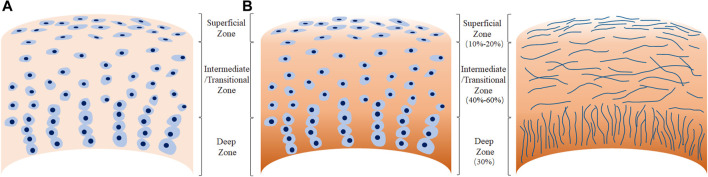
Morphology of human adult articular cartilage. **(A)**, chondrocytes organization in the different tissue zones. **(B)**, arrangement of collagen fibers.

The ECM comprises of a fibrillary network of collagen, non-collagenous proteins, proteoglycans, and water; and the orientation of the collagen fibrils along with chondrocyte morphology characterize the three zones of AC (Figure 1). In the thin superficial zone, that makes up approximately 10–20% of the AC thickness, the collagen fibrils and chondrocytes are aligned parallel to the articular surface. Chondrocytes are flattened and elongated, and the integrity of this layer is vital for the protection of the deeper layers. This zone confers the tensile properties to the AC, and helps resist the sheer forces experienced by the joint. The middle layer or the transitional zone which makes up 40–60% of the AC thickness is characterized by obliquely organized thicker collagen fibrils, and slightly larger chondrocytes at a relatively lower density. The transitional zone is the first line of resistance to the compressive forces imposed by the articulation. In the deep zone, the collagen fibrils are the thickest and are arranged perpendicular to the articulating surfaces. The very large chondrocytes are typically arranged in columns, parallel to the collagen fibrils. This deep zone which make up nearly 30% of the AC volume, provides the greatest resistance to the compressive forces (Ulrich-Vinther et al., 2003). Calcified cartilage, which lies beneath the deep zone separated by the tide mark, secures the AC by anchoring the collagen fibrils to the subchondral bone (Sophia Fox et al., 2009).

There are three main types of cartilages found in the human body, with differing structures and functions.

### Hyaline Cartilage

Hyaline cartilage is the most common type of cartilage and can be seen at the connection sites between the ribs and sternum, trachea, and on the surfaces of the synovial joints. It has a glassy appearance and type II collagen is the most abundant type of collagen in hyaline cartilage, accounting for 90–95% of the collagen in ECM. Additionally, collagen IX links covalently to the type II collagen backbone, with type XI collagen forming the filamentous template of the fibril (Eyre et al., 2006). The matrix of hyaline cartilage is also rich in glycosaminoglycans (GAGs), including negatively charged hyaluronic acid and chondroitin sulfate. Aggrecan (ACAN), is a major proteoglycan in the AC, which interacts with the GAGs to form large aggregates and their high anionic charge allow for increased retention of water molecules thus facilitating shock absorption. In adults, chondrocytes show low anabolic and proliferative activity, and the collagen fibers last life-long, with minimal replacement activity.

### Fibrocartilage

Fibrocartilage is mainly found between the vertebral bodies, symphysis pubis, meniscus, and tendon-bone interface. Its matrix is rich in densely braided collagen fibers, making it highly resistant to compressive loads. The chondrocytes are aligned with the thick collagen fibers, and are very few in number. Compared to hyaline cartilage, fibrocartilage contains a large amount of type I collagen in addition to type II collagen (Armiento et al., 2019).

### Elastic Cartilage

Elastic cartilage is a type of elastic and flexible tissue that can withstand repeated bending and is found in epiglottis, auricle and eustachian tube. In elastic cartilage, the cell morphology is basically similar to that of hyaline cartilage. Type II collagen and elastic fibers branch densely in multiple directions, and contains relatively low amounts of type III, XII, V collagen (Wachsmuth et al., 2006).

## The Role of Microenvironment on Cartilage Function

Considering the avascular characteristic of AC, chondrocyte metabolism, and thus cartilage homeostasis largely depends on the diffusion of nutrients, oxygen, and other regulatory factors from subchondral bone and synovial fluid through the matrix. In this section, we discuss the current known factors in the chondrocyte microenvironment that regulate cartilage homeostasis and function, with specific focus on mechanical stimulation, oxygen tension, and cytokines and growth factors.

### Mechanical Stimulation

**FIGURE 2:**
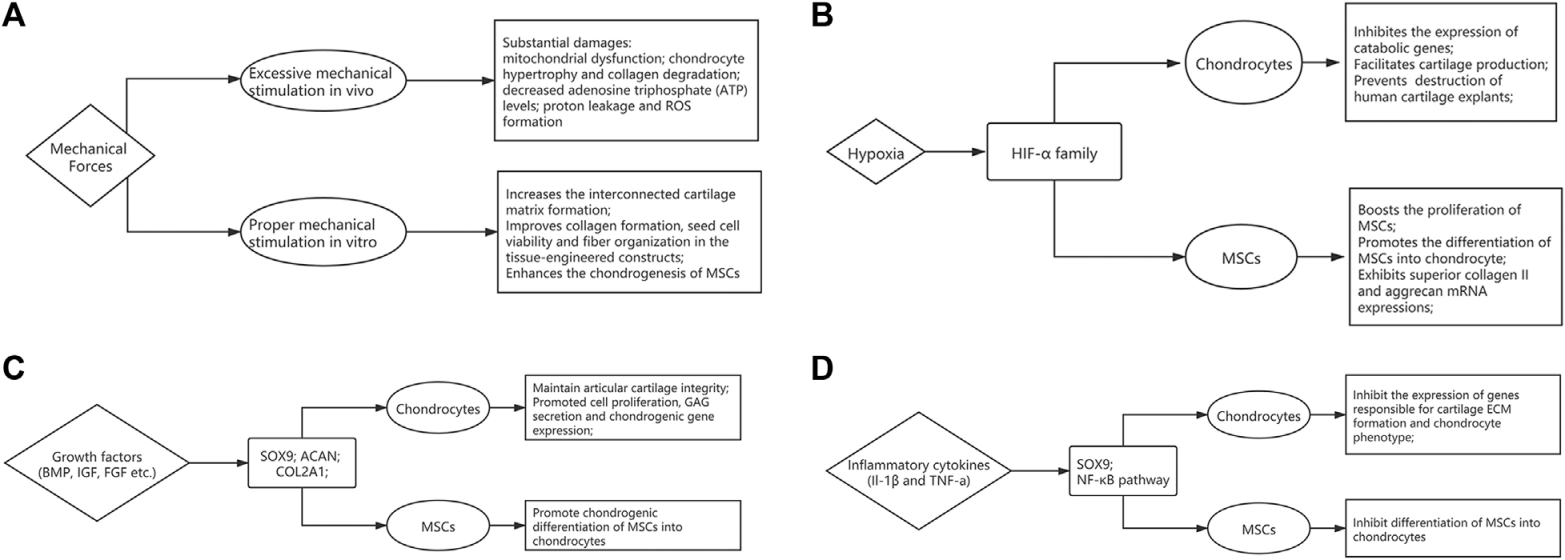
The influence of microenvironmental factors on articular cartilage homeostasis and function. The flow-chart describes the effects of **(A)** mechanical forces, **(B)** Oxygen tension, **(C)** growth factors and **(D)** inflammatory cytokines.

The chondrocytes reside in a microenvironment that experiences complex combination of compressive loads, shear, and tensile stress regularly. Sensitivity to such mechanical stimuli and consequent adaptive responses are essential to maintain the joint function. Optimal mechanical stress has been shown to induce chondrogenesis during fetal development and maintains cartilage homeostasis in adults (Zhao et al., 2020). However, impact forces caused by falls, sports injuries and road accidents can lead to substantial damage. Indeed, studies have shown that above a certain threshold, impact forces can cause permanent changes to the mechanical properties and damage the structure of AC (Jeffrey and Aspden, 2006; Verteramo and Seedhom, 2007). Additionally, repetitive heavy loading has been shown to cause chondrocyte mitochondrial dysfunction, decreasing adenosine triphosphate (ATP) levels, exacerbating proton leakage and ROS formation (Coleman et al., 2016). Transforming growth factor-β (TGF- β) signaling is a well-established mediator of mechanical stress based regulation of cartilage. In a recent study, Zhen et al. show that TGF-β activity is concentrated in areas of cartilage that experience high mechanical stress, and it impairs the metabolic activity of chondrocytes, thus disrupting cartilage homeostasis (Zhen et al., 2021). Other pathways including MAPK-ERK, Wnt and Indian hedgehog signaling have been shown to play a role in regulating mechanotransduction in chondrocytes, and have been reviewed elsewhere (Zhao et al., 2020) (Figure 2A).

The insights of mechanobiology can provide new strategies in regenerative medicine. In order to mimic the natural environment of AC, mechanical stimulation can be added during expansion cultures of cell-based therapies (see section 4.2). At the cellular level, using biomechanics to enhance the chondrogenesis of mesenchymal stem cells has been well documented, which could be attributable to increased TGF-β meditated chondrogenic signaling (Li et al., 2010; Fahy et al., 2018). Low level of shear stress has shown to stimulate hMSC migration through JNK and p38 MAPK pathways mediated by SDF-1/CXCR4 axis (Yuan et al., 2013). In addition, mechanical loading enhances the angiogenic capacity, which could be attributed to FGFR and VEGFR signaling cascades (Kasper et al., 2007). Furthermore, optimal mechanical stimulation also has the potential to improve collagen formation, cell viability and fiber organization in the tissue-engineered constructs (Li et al., 2017; Salinas et al., 2018).

### Oxygen Tension

AC microenvironment is generally hypoxic due to the lack of capillary network, and several studies have highlighted the relationship between hypoxia and AC development and homeostasis. The oxygen tension in the AC may be as low as 1–3% compared to 21% under normoxic conditions. Hypoxic environment has long been acknowledged as a positive influence that drives cartilage matrix accumulation, supporting a healthy chondrocyte phenotype (Murphy et al., 2009). Hypoxia-inducible factors (HIFs) are essential regulators that respond to hypoxia. They consist of an oxygen-regulatory α subunit, and a constitutively expressed β subunit. Under normal oxygen tension, the α subunit is degraded following hydroxylation by prolyl hydroxylases. However, it is stabilized under hypoxic conditions, dimerizes with the β subunit, and translocates to the nucleus to regulate gene expression. HIF-1α has been shown to be essential for chondrocyte survival and development of murine growth plates (Schipani et al., 2001). Furthermore, hypoxia has been associated with upregulation in expression of matrix components, and downregulation of degrading enzymes and hypertrophic markers in both healthy and OA chondrocytes (Murphy and Polak, 2004; Markway et al., 2013). A recently published study demonstrated that HIF-1α has anti-catabolic effects on AC by inhibiting the expression of Mmp13, via suppression of NF-κB signaling (Okada et al., 2020). Furthermore, using RNA interference, HIF-2α and not HIF-1α, was identified as a critical anabolic regulator of matrix synthesis (Lafont et al., 2007). It was also demonstrated that matrix genes such as aggrecan and type IX collagen were not HIF targets but regulated by hypoxia via SOX9, a cartilage-specific transcription factor (Thoms et al., 2013) (Figure 2B).

Several studies have highlighted the role of hypoxia in chondrogenic differentiation of mesenchymal stem cells (MSCs) (Robins et al., 2005; Elabd et al., 2018; Contentin et al., 2020). Proliferation of bone marrow MSCs was enhanced under hypoxia, together with increased chondrogenic ability, and higher type II collagen and aggrecan mRNA expressions (Bornes et al., 2015). Furthermore, hypoxia mediated HIF-1α stabilization led to activation of SOX9 and subsequent differentiation of MSCs to chondrocytes (Robins et al., 2005). Thus, targeting the hypoxic pathways, possibly by inhibiting prolyl hydroxylases (Joharapurkar et al., 2018), could be therapeutic strategy for cartilage repair.

### Cytokines and Growth Factors

During development, the synthesis of matrix components by chondrocytes is stimulated by a range of anabolic cytokines including TGF-β (Wang et al., 2014), bone morphogenetic proteins (BMPs) (Kobayashi et al., 2005) and insulin-like growth factor-1 (IGF-1) (Lui et al., 2019). The maintenance of cartilage health requires the bioactive levels of TGF-β to be within a narrow range. Concentrations above or below this optimal range and subsequent alterations in the TGF-β/SMAD2/3 pathway result in abnormal cartilage function (Finnson et al., 2012; Zhai et al., 2015). High concentrations of TGF-β induced by mechanical stress and platelet-derived growth factor-BB (PDGF-BB) secreted by mononuclear preosteoclasts respectively, cause bone resorption and angiogenesis in subchondral bone. Since subchondral bone is crucial for maintaining AC integrity, pathological changes in subchondral bone exacerbate the progression of OA (Zhen et al., 2013; Su et al., 2020). BMPs have been shown to regulate several chondrocyte specific genes and are involved all phases of chondrogenesis. *In vitro* studies have highlighted the role of BMP-7 in chondrocyte proliferation, GAG secretion and chondrogenic gene expression including ACAN, SOX9 and Col2a1 (Shen et al., 2010). BMP-2 mediated induction of chondrocyte differentiation from MSCs has been shown to occur via TGF-β3 pathway (Shen et al., 2009). BMP-6 has also been shown to promote chondrogenesis and hypertrophic differentiation from MSCs (Sekiya et al., 2001), and stimulate matrix synthesis (Bobacz et al., 2003). IGF-1 also modulates chondrogenesis from MSCs by stimulating cell proliferation, regulating apoptosis, and inducing chondrogenic gene expression (Longobardi et al., 2006; Ikeda et al., 2017). Moreover, mechanical stimulation has a synergistic effect on IGF-1 mediated increase in collagen and proteoglycan synthesis (Bonassar et al., 2001). Furthermore, chronic deficiency in IGF-1 levels has shown to associate with increased severity of AC lesions in OA in a rat model (Ekenstedt et al., 2006) (Figure 2C).

In response to trauma or inflammatory disease such as OA, a process of cartilage remodeling initiates. A catabolic response is mediated by inflammatory cytokines interleukin-1 (IL-1) and tumor necrosis factor-a (TNF-α) which suppress Sox9 mRNA and protein levels via the NF-κB pathway. This leads to a marked inhibition in expression of cartilage specific genes responsible for ECM formation and chondrogenesis (Murakami et al., 2000). This was confirmed in a separate study which showed IL-1β and TNF-α mediated inhibition of chondrogenesis by human MSCs through NF-κB dependent mechanisms (Wehling et al., 2009). IL-1β has also been shown to upregulate the expression of matrix metalloproteinase-3 (MMP3) and tumor-necrosis-factor-stimulated gene 6 (TSG6), and downregulate ACAN, further exacerbating the catabolic phenotype (Stöve et al., 2000). TNF-α also induces the expression of cartilage degradation molecules including MMP-9 and MMP-13, and decreases collagen type II and type XI synthesis (Lefebvre et al., 1990; Reginato et al., 1993; Liacini et al., 2003) (Figure 2D).

## Current and Emerging Treatment Modalities

Articular cartilage lesions, when left untreated, leads to the onset of degenerative OA, and thus demands an effective treatment option for repair and regeneration. Here we discuss the current treatment modalities used for the repair for cartilage lesions, and the emerging technologies.

### Surgical Approaches

Surgical approaches for articular cartilage can be divided into three categories: bone marrow stimulation, autografts and allografts, and cell-based therapies. Bone marrow stimulation procedures are widely applied in treatment of osteochondral lesions treatment, which include microfracture, subchondral drilling or abrasion of the subchondral bone. Microfracture generally targets small, contained defects and requires making several perforations in the subchondral plate by using an arthroscopic awl and may result in good clinical outcome for smaller lesions (Sommerfeldt et al., 2016). Nevertheless, microfracture might ultimately induce the formation of fibrocartilage since bone marrow released into the defect forms a blood clot (Goyal et al., 2013; Kwon et al., 2019). Subchondral drilling and subchondral abrasion are alternatives to microfracture, but are less popular due to the risks of thermal necrosis, hypertrophy, or cysts (Chen et al., 2009).

Osteochondral autograft directly delivers mature and viable hyaline cartilage into the defects and therefore realizes a faster rehabilitation compared to most other cartilage repair strategies (Redondo et al., 2018). However, the application of osteochondral autograft transfer is restricted to small chondral defects (<2 cm^2^) because of the limitations in graft availability (Hangody et al., 2004). Osteochondral allograft transplantation is a feasible solution for AC repair due to the avascular nature of cartilage which alleviates any immunological concerns (Arzi et al., 2015). Although osteochondral allograft transplantation overcomes the graft availability concerns of osteochondral autograft transfer, the difficulties in matching allografts to native architecture and improving the viability and longevity of the fresh tissue still exists (Koh et al., 2006; Cook et al., 2016).

Autologous chondrocyte implantation (ACI) is the most widely used surgical approach to treat large chondral defects (>3–4 cm^2^), and is the first application of cell engineering strategy in orthopedic surgery. It relies on repopulating the cartilage lesion with mature autologous chondrocytes, and thus requires two surgeries–one to harvest chondrocytes from healthy cartilage, and another to reimplant them into defects after expansion in cultures *in vitro* (Welch et al., 2016). Approximately 22% of patients suffer from hypertrophy of the periosteum after ACI procedures (Gikas et al., 2009). Matrix-induced ACI (MACI) is the second generation of this technique which involves incorporation of chondrocytes onto a collagen scaffold prior to implantation (Nawaz et al., 2014). Although both ACI and MACI show better hyaline or hyaline-like cartilage formation and graft survival, patients have to endure two surgeries, a longer recovery phase and high financial costs (Chimutengwende-Gordon et al., 2020). Furthermore, the chondrocyte can de-differentiate during culture expansion, with downregulation of chondrogenic markers resulting in a limited life-span following implantation (Brittberg et al., 2003).

### Cell Based Therapy

Chondrocytes often lose their capacity to produce ECM and proliferate after passaging in culture, a phenomenon referred to as de-differentiation (Goldring et al., 1986; Schnabel et al., 2002). A recent study identified an important role of perlecan, a heparen sulphate proteoglycan, in the repair of defects in human cartilage. The authors also demonstrate that treating the chondrocytes with heperanase increased their proliferative potential and chondrogenic gene expression, with implications for *in vitro* expansion of cells (Garcia et al., 2021). A new population of cells termed as cartilage-derived stem/progenitor cells (CPSCs) have been identified in the cartilage, which unlike the chondrocytes, may have the ability to self-renew (Jiang and Tuan, 2015). The application potential of CPSCs is still unclear, but research is ongoing to better understand this cell phenotype and its therapeutic prospects for AC repair (Jiang et al., 2016; Bauza et al., 2020).

MSCs offer a promising cell source for regeneration and repair of cartilage lesions, as they have the ability to differentiate into chondrocytes, and are easy to harvest, with minimal donor site morbidity (Park et al., 2018). MSCs used for repairing the cartilage lesions are obtained from a variety of autologous tissues including bone marrow (BM-MSCs), adipose tissue (AT-MSCs) and peripheral blood (PB-MSCs) (Reissis et al., 2016). Depending on the specific cartilage pathology, the MSCs can either be implanted into the defect following surgical incision, or administered via intra-articular injection. A post-surgical prognosis study assessing the efficacy of AT-MSCs implantation for cartilage lesions observed 76% of the patients had the repair rated as abnormal or severely abnormal as per the International Cartilage Repair Society standards (Koh et al., 2014). Furthermore, compared to MSCs implantation, intra-articular injections have a higher risk of the cells migrating to non-target tissues (Reissis et al., 2016). Whilst, there have been several studies which have reported improved outcomes following MSC based therapies (Wakitani et al., 2002; Lee et al., 2019) there are some challenges that need to be overcome.

Indeed, multiple novel strategies are being explored to improve the use MSC in cartilage regeneration. For instance, a magnetically actuated microrobot has recently been proposed to improve the targeting efficiency of MSC in tissue regeneration for cartilage lesions (Go et al., 2020). Other studies are exploiting the ability of MSCs to exert their regenerative functions by secreting paracrine molecules that modulate the local immune response or improves chondrocytes cell survival and proliferation (Toh et al., 2017; Zhang et al., 2019; Kim et al., 2020). By using MSCs derived extracellular vesicles, studies have shown improved cartilage regeneration and repair (Vonk et al., 2018; Alcaraz et al., 2019).

### Small Molecules

A variety of synthetic or natural small molecule compounds have proven effective in maintaining a stable chondrocyte phenotype, supporting an ideal microenvironment to promote chondrocyte proliferation and chondrogenic differentiation of stem cells (Li et al., 2020). Kartogenin (KGN) can facilitate chondrocyte differentiation by regulating the CBFβ-RUNX1 signaling pathway in BM-MSCs (Johnson et al., 2012). Moreover, KGN can enhance the therapeutic effect of conventional treatment modalities. Intra-articular injection of KGN after microfracture showed increased hyaline-like cartilage formation and better defect filling, indicating an enhanced quality of repair for full-thickness cartilage defects (Xu et al., 2015).

Another small molecule compound TD-198946, a derivative of thienoindazole, can strongly induce chondrogenic differentiation via upregulation of Runx1 expression, preventing and repairing degeneration of the articular cartilage (Yano et al., 2013). A novel synthetic small molecule 5{i,2} was discovered to induce a more directed chondrogenic differentiation of BM-MSCs compared to TGF-β3. Molecule 5{i,2} is thought to exert its effect by enhancing the transcription of chondrogenic markers including SOX9 and aggrecan (Cho et al., 2012). A small molecule called BNTA was described to target superoxide dismutase 3 (SOD3) in the cartilage microenvironment and induce superoxide anion elimination in chondrocyte culture, promoting generation of ECM components (Shi et al., 2019). Salidroside, isolated from the root of *Rhodiola rosea*, improves chondrocyte proliferation, prevents apoptosis, and drives cartilage specific gene expression via TGF-β/Smad3 and PI3K/Akt signaling pathways (Zhang and Zhao, 2018; Wu et al., 2019). Lastly, berberine chloride, derived from *Coptis chinensis* and *Berberis aristata*, has been verified to stimulate chondrocyte proliferation and upregulate aggrecan and Col II expression through activating Wnt/β-catenin pathways (Zhou et al., 2016).

Such small molecule drugs present an attractive therapeutic strategy for MSC based cartilage regeneration and repair. They have a significant cost advantage compared to biologics, more predictable pharmacokinetic and pharmacodynamic profile and are usually orally bioavailable, thus making them a convenient option (Ngo and Garneau-Tsodikova, 2018).

### Tissue Engineering

Tissue engineering is using a combination of cells, biochemical and physio-chemical factors along with engineering and biomaterials to improve or replace biological function (Musumeci et al., 2014). Tissue engineering in cartilage repair can be divided into two categories, scaffold-dependent and independent approaches. Scaffold refers to a variety of synthetic or natural biomaterials which construct a suitable environment for cell distribution, cell–matrix and cell–cell interactions (Kwon et al., 2019). In addition, an ideal biomaterial scaffold for AC regeneration can now be bioactive, biomimetic, biodegradable and bioresponsive, allowing signaling with spatio-temporal control (Musumeci et al., 2014). Scaffolds can be made of a wide array of materials including polyglycolides, polylactides, silk and decellularized cartilage-derived matrix (Kwon et al., 2019). Hydrogel-based scaffolds are becoming one of the most prevalent treatment strategies in cartilage defect repair. Injectable hydrogels can form irregular shapes that better fill the cartilage defects, provide a high-water content scaffold similar to natural ECM and are minimally invasive (Liu et al., 2017). More recently, injectable thermogels formed by the sol-gel phase transition have been employed to repair cartilage defects. A thermogel of triblock copolymer poly (lactide-co-glycolide)-block-poly (ethyleneglycol)-block-poly (lactide-co-glycolide) (PLGA−PEG−PLGA) dissolves in water at low temperatures, but gels spontaneously at body temperatures. In a rabbit model of full-thickness cartilage defect, the BM-MSC incorporated PLGA−PEG−PLGA thermogel regenerated cartilage with high expression of GAGs and type II collagen, and comparable biomechanical properties to normal cartilage (Zhang et al., 2018).

While scaffolds provide an initial mechanical stability, it has several limitations including degradation-associated toxicity, hindrances to remodelling, stress shielding, and altered cell phenotypes (Athanasiou et al., 2013). Therefore, investigations were directed into developing scaffold-free strategies. Three dimensional (3D) bioprinting is the placing of cells in a 3D space to generate a cohesive tissue microarchitecture akin to the *in vivo* characteristics (Moldovan et al., 2019). Hydrogels are fast becoming a common printing media which are then jetted with cells, an approach referred to as bio-ink based hybrid bioprinting (Laternser et al., 2018; Moldovan et al., 2019). Hydrogels can differ in their physical properties including viscosity and rigidity, which has implications for the mechanical environment, and the amount of natural cell binding motifs. This may in turn influence cell spreading and cell–matrix interactions. Therefore, the choice of bioink is crucial considering the chondrocyte phenotype varies in fibrocartilage and hyaline cartilage (Daly et al., 2016). In addition, the scaffold free cell-sheet engineering wherein 3D MSC sheets are developed to create a transplantable hyaline-like cartilage tissue have successfully shown chondrogenic differentiation capability (Thorp et al., 2020).

At present, most of the research on the role of tissue microenvironment on regulating AC and chondrocyte phenotype rely on *in vitro* experiments, and requires further exploration *in vivo*. Better understanding of the influence of the microenvironment on chondrocytes and MSCs can provide vital insights for development of novel therapies. For instance, the use of chondrogenic growth factors in scaffolds can improve cartilage synthesis and improve implantation outcomes (Chen et al., 2020). The application of platelet-rich plasma (PRP) in cartilage repair is an excellent example of combining the understanding of molecular biology with regenerative medicine to treat cartilage injury (Abrams et al., 2013; Kennedy et al., 2018). PRP consists of concentrated platelets, white blood cells, plasma, and various other growth factors. On one hand, PRP can be used as a natural scaffold for tissue engineering (Wu et al., 2009; Vinod et al., 2019). Once activated, PRP acts as a gelatinous scaffold that supports the growth of seeded cells and lubricates the articular cartilage, reducing the coefficient of friction and wear. On the other hand, PRP also contains growth factors that can directly promote cartilage repair. Studies have shown that PRP can regulate synovial inflammation and reduce pain through the inflammatory NF-κB signaling pathway. PRP also improves the expression of TGF-β, aggregan, and type II collagen, thus promoting cell migration, proliferation, and differentiation of progenitor/stem cells (Mascarenhas et al., 2015; Sakata and Reddi, 2016; Moussa et al., 2017). Therefore, PRP has potential applications in improving the efficacy of traditional surgical treatment, cell therapy, and tissue engineering.

## Conclusion and Future Outlook

Articular cartilage regeneration and repair is a dynamic multidisciplinary field that is continuously evolving. The current clinical approaches have achieved limited success, however, the rapid advances in cell-based therapies, biomaterials, and developments in mechanobiology have the potential to provide long-term solutions for cartilage repair and regeneration. Comprehensive treatment modalities with lower financial costs, shorter recovery period, and reduced surgical recovery is expected in the future.

The formation of fibrocartilage remains a major obstacle in cartilage repair. Fibrosis is characterized by activation and proliferation of fibroblasts or fibrogenic cells, accompanied by an over deposition of ECM (Novo et al., 2009; Sakai and Tager, 2013). In case of AC, the repair procedures after cartilage injury may lead to excessive secretion and deposition of ECM proteins, which may result in fibrocartilage formation (Pearle et al., 2005; Chan et al., 2018). In addition, the inadequate number of stem cells and progenitor cells recruited to the injured site after cartilage damage or microfracture surgery can exacerbate the formation of fibrocartilage (Im, 2016; Hu et al., 2021). The fundamental objective for treating cartilage injury remains the regeneration of hyaline cartilage, or transplant mature hyaline cartilage tissue. However, the potential reparative role of fibrocartilage has been largely neglected due to its inferior mechanical properties. Preventing hyaline cartilage fibrosis and promoting the hyalinization of fibrocartilage may provide new therapeutic ideas for repairing cartilage damage (Shi and Li, 2021).

There are two strategies that could be employed to prevent hyaline cartilage fibrosis. The first is to reshape the microenvironment of the injury site. By adding appropriate cytokines, the spontaneous inflammatory response after injury can be alleviated and cell proliferation and differentiation can be promoted. The three-dimensional structure and physical properties of the grafts used in ACI and MACI could also be optimized to closely simulate the structure of articular cartilage, display better response to mechanical pressure and other stimuli, and therefore be more suitable for the growth of resident cells. The avascular nature of AC makes it difficult for nutrients to diffuse, and the ECM of AC is relatively dense. These factors contribute to the difficulty for mesenchymal stem cells and progenitor cells to migrate to injury sites. The second strategy could be to promote migration of the endogenous skeletal or mesenchymal stem/progenitor cells to the AC. Application of chemoattractants could facilitate this migration of resident mesenchymal stem/progenitor cells, and thus improve the repair capacity (Dwyer et al., 2007; Park et al., 2015; Wang et al., 2017).

Finally, in order to achieve fibrocartilage hyalinization, it is necessary to identify where the cells within the fibrosis tissue come from. Studies have shown that chondrocytes originated from synovium-derived mesenchymal stem cells and articular chondrocytes have similar gene expression profiles, suggesting that synovial and AC are formed from the same precursors (Zhou et al., 2014; Caldwell and Wang, 2015). This may explain why repair of cartilage damage with synovium-derived mesenchymal stem cells shows less de-differentiation and fibrosis, and thus better outcomes in some cases than with other types of mesenchymal stem cells (Sakaguchi et al., 2005; Pei and He, 2012). However, the origins of fibrocartilage after injury still remain unclear. In addition, changes in cell behavior in fibrotic tissue (such as cytoskeletal activity, cell metabolism, etc.), and alterations in gene expression at transcriptional and translational levels need to be clarified. This is essential to thoroughly explore the mechanism of fibrocartilage formation and screen suitable stimulus factors and small molecule drugs that can modify the microenvironment to minimize the fibrocartilage phenotype and promote hyalinization of the cartilage.

## References

[B1] Abrams G. D. Frank R. M. Fortier L. A. Cole B. J. (2013). Platelet-rich Plasma for Articular Cartilage Repair. Sports Med. Arthrosc. Rev. 21, 213–219. 10.1097/JSA.0b013e3182999740 24212369

[B2] Alcaraz M. J. Compañ A. Guillén M. I. (2019). Extracellular Vesicles from Mesenchymal Stem Cells as Novel Treatments for Musculoskeletal Diseases. Cells 9, 98. 10.3390/cells9010098 PMC7017209 31906087

[B3] Armiento A. R. Alini M. Stoddart M. J. (2019). Articular Fibrocartilage - Why Does Hyaline Cartilage Fail to Repair? Adv. Drug Deliv. Rev. 146, 289–305. 10.1016/j.addr.2018.12.015 30605736

[B4] Arzi B. Duraine G. D. Lee C. A. Huey D. J. Borjesson D. L. Murphy B. G. (2015). Cartilage Immunoprivilege Depends on Donor Source and Lesion Location. Acta Biomater. 23, 72–81. 10.1016/j.actbio.2015.05.025 26028293 PMC4522233

[B5] Athanasiou K. A. Eswaramoorthy R. Hadidi P. Hu J. C. (2013). Self-organization and the Self-Assembling Process in Tissue Engineering. Annu. Rev. Biomed. Eng. 15, 115–136. 10.1146/annurev-bioeng-071812-152423 23701238 PMC4420200

[B6] Bauza G. Pasto A. Mcculloch P. Lintner D. Brozovich A. Niclot F. B. (2020). Improving the Immunosuppressive Potential of Articular Chondroprogenitors in a Three-Dimensional Culture Setting. Sci. Rep. 10, 16610. 10.1038/s41598-020-73188-9 33024130 PMC7538570

[B7] Bobacz K. Gruber R. Soleiman A. Erlacher L. Smolen J. S. Graninger W. B. (2003). Expression of Bone Morphogenetic Protein 6 in Healthy and Osteoarthritic Human Articular Chondrocytes and Stimulation of Matrix Synthesis *In Vitro* . Arthritis Rheum. 48, 2501–2508. 10.1002/art.11248 13130469

[B8] Bonassar L. J. Grodzinsky A. J. Frank E. H. Davila S. G. Bhaktav N. R. Trippel S. B. (2001). The Effect of Dynamic Compression on the Response of Articular Cartilage to Insulin-like Growth Factor-I. J. Orthop. Res. 19, 11–17. 10.1016/s0736-0266(00)00004-8 11332605

[B9] Bornes T. D. Jomha N. M. Mulet-Sierra A. Adesida A. B. (2015). Hypoxic Culture of Bone Marrow-Derived Mesenchymal Stromal Stem Cells Differentially Enhances *In Vitro* Chondrogenesis within Cell-Seeded Collagen and Hyaluronic Acid Porous Scaffolds. Stem Cel Res Ther 6, 84. 10.1186/s13287-015-0075-4 PMC4431536 25900045

[B10] Borrelli J. Jr. Olson S. A. Godbout C. Schemitsch E. H. Stannard J. P. Giannoudis P. V. (2019). Understanding Articular Cartilage Injury and Potential Treatments. J. Orthop. Trauma 33 (6), S6–s12. 10.1097/bot.0000000000001472 31083142

[B11] Brittberg M. Peterson L. Sjögren-jansson E. Tallheden T. Lindahl A. (2003). Articular Cartilage Engineering with Autologous Chondrocyte Transplantation. The J. Bone Jt. Surgery-American. 85 (3), 109–115. 10.2106/00004623-200300003-00017 12925617

[B12] Caldwell K. L. Wang J. (2015). Cell-based Articular Cartilage Repair: the Link between Development and Regeneration. Osteoarthritis and Cartilage 23, 351–362. 10.1016/j.joca.2014.11.004 25450846 PMC4339504

[B13] Chan D. D. Li J. Luo W. Predescu D. N. Cole B. J. Plaas A. (2018). Pirfenidone Reduces Subchondral Bone Loss and Fibrosis after Murine Knee Cartilage Injury. J. Orthop. Res. 36, 365–376. 10.1002/jor.23635 28646530 PMC5742076

[B14] Chen H. Sun J. Hoemann C. D. Lascau-Coman V. Ouyang W. Mckee M. D. (2009). Drilling and Microfracture lead to Different Bone Structure and Necrosis during Bone-Marrow Stimulation for Cartilage Repair. J. Orthop. Res. 27, 1432–1438. 10.1002/jor.20905 19402150

[B15] Chen L. Liu J. Guan M. Zhou T. Duan X. Xiang Z. (2020). Growth Factor and its Polymer Scaffold-Based Delivery System for Cartilage Tissue Engineering. Ijn 15, 6097–6111. 10.2147/ijn.S249829 32884266 PMC7434569

[B16] Chiang H. Jiang C.-C. (2009). Repair of Articular Cartilage Defects: Review and Perspectives. J. Formos. Med. Assoc. 108, 87–101. 10.1016/s0929-6646(09)60039-5 19251544

[B17] Chimutengwende-Gordon M. Donaldson J. Bentley G. (2020). Current Solutions for the Treatment of Chronic Articular Cartilage Defects in the Knee. EFORT Open Rev. 5, 156–163. 10.1302/2058-5241.5.190031 32296549 PMC7144889

[B18] Cho T.-J. Kim J. Kwon S.-K. Oh K. Lee J.-A. Lee D.-S. (2012). A Potent Small-Molecule Inducer of Chondrogenic Differentiation of Human Bone Marrow-Derived Mesenchymal Stem Cells. Chem. Sci. 3, 3071–3075. 10.1039/C2SC20362F

[B19] Chubinskaya S. Haudenschild D. Gasser S. Stannard J. Krettek C. Borrelli J. Jr. (2015). Articular Cartilage Injury and Potential Remedies. J. Orthop. Trauma 29 (12), S47–S52. 10.1097/bot.0000000000000462 26584267 PMC7054985

[B20] Coleman M. C. Ramakrishnan P. S. Brouillette M. J. Martin J. A. (2016). Injurious Loading of Articular Cartilage Compromises Chondrocyte Respiratory Function. Arthritis Rheumatol. 68, 662–671. 10.1002/art.39460 26473613 PMC4767543

[B21] Contentin R. Demoor M. Concari M. Desancé M. Audigié F. Branly T. (2020). Comparison of the Chondrogenic Potential of Mesenchymal Stem Cells Derived from Bone Marrow and Umbilical Cord Blood Intended for Cartilage Tissue Engineering. Stem Cel Rev Rep 16, 126–143. 10.1007/s12015-019-09914-2 31745710

[B22] Cook J. L. Stannard J. P. Stoker A. M. Bozynski C. C. Kuroki K. Cook C. R. (2016). Importance of Donor Chondrocyte Viability for Osteochondral Allografts. Am. J. Sports Med. 44, 1260–1268. 10.1177/0363546516629434 26920431

[B23] Correa D. Lietman S. A. (2017). Articular Cartilage Repair: Current Needs, Methods and Research Directions. Semin. Cel Developmental Biol. 62, 67–77. 10.1016/j.semcdb.2016.07.013 27422331

[B24] Daly A. C. Critchley S. E. Rencsok E. M. Kelly D. J. (2016). A Comparison of Different Bioinks for 3D Bioprinting of Fibrocartilage and Hyaline Cartilage. Biofabrication 8, 045002. 10.1088/1758-5090/8/4/045002 27716628

[B25] Dwyer R. M. Potter-Beirne S. M. Harrington K. A. Lowery A. J. Hennessy E. Murphy J. M. (2007). Monocyte Chemotactic Protein-1 Secreted by Primary Breast Tumors Stimulates Migration of Mesenchymal Stem Cells. Clin. Cancer Res. 13, 5020–5027. 10.1158/1078-0432.Ccr-07-0731 17785552

[B26] Ekenstedt K. J. Sonntag W. E. Loeser R. F. Lindgren B. R. Carlson C. S. (2006). Effects of Chronic Growth Hormone and Insulin-like Growth Factor 1 Deficiency on Osteoarthritis Severity in Rat Knee Joints. Arthritis Rheum. 54, 3850–3858. 10.1002/art.22254 17133593

[B27] Elabd C. Ichim T. E. Miller K. Anneling A. Grinstein V. Vargas V. (2018). Comparing Atmospheric and Hypoxic Cultured Mesenchymal Stem Cell Transcriptome: Implication for Stem Cell Therapies Targeting Intervertebral Discs. J. Transl Med. 16, 222. 10.1186/s12967-018-1601-9 30097061 PMC6086019

[B28] Eyre D. Weis M. A. Weis M. Wu J.-J. (2006). Articular Cartilage Collagen: an Irreplaceable Framework? eCM 12, 57–63. 10.22203/ecm.v012a07 17083085

[B29] Fahy N. Alini M. Stoddart M. J. (2018). Mechanical Stimulation of Mesenchymal Stem Cells: Implications for Cartilage Tissue Engineering. J. Orthop. Res. 36, 52–63. 10.1002/jor.23670 28763118

[B30] Finnson K. W. Chi Y. Bou-Gharios G. Leask A. Philip A. (2012). TGF-beta Signaling in Cartilage Homeostasis and Osteoarthritis. Front. Biosci. S4, 251–268. 10.2741/s26610.2741/266 22202058

[B31] Garcia J. Mccarthy H. S. Kuiper J. H. Melrose J. Roberts S. (2021). Perlecan in the Natural and Cell Therapy Repair of Human Adult Articular Cartilage: Can Modifications in This Proteoglycan Be a Novel Therapeutic Approach? Biomolecules 11, 92. 10.3390/biom11010092 33450893 PMC7828356

[B32] Gikas P. D. Bayliss L. Bentley G. Briggs T. W. R. (2009). An Overview of Autologous Chondrocyte Implantation. J. Bone Jt. Surg. Br. 91-B, 997–1006. 10.1302/0301-620x.91b8.21824 19651824

[B33] Go G. Jeong S.-G. Yoo A. Han J. Kang B. Kim S. (2020). Human Adipose–Derived Mesenchymal Stem Cell–Based Medical Microrobot System for Knee Cartilage Regeneration *In Vivo* . J. Sci. Robotics 5, eaay6626. 10.1126/scirobotics.aay6626 33022593

[B34] Goldring M. B. Sandell L. J. Stephenson M. L. Krane S. M. (1986). Immune Interferon Suppresses Levels of Procollagen mRNA and Type II Collagen Synthesis in Cultured Human Articular and Costal Chondrocytes. J. Biol. Chem. 261, 9049–9055. 10.1016/s0021-9258(19)84486-1 3087985

[B35] Goyal D. Keyhani S. Lee E. H. Hui J. H. P. (2013). Evidence-based Status of Microfracture Technique: a Systematic Review of Level I and II Studies. Arthrosc. J. Arthroscopic Relat. Surg. 29, 1579–1588. 10.1016/j.arthro.2013.05.027 23992991

[B36] Hangody L. Ráthonyi G. K. Duska Z. Vásárhelyi G. Füles P. Módis L. (2004). Autologous Osteochondral Mosaicplasty. J. Bone Jt. Surgery-American. 86, 65–72. 10.2106/00004623-200403001-00009 14996923

[B37] Hu H. Liu W. Sun C. Wang Q. Yang W. Zhang Z. (2021). Endogenous Repair and Regeneration of Injured Articular Cartilage: A Challenging but Promising Therapeutic Strategy. Aging Dis. 12, 886–901. 10.14336/ad.2020.0902 34094649 PMC8139200

[B38] Ikeda Y. Sakaue M. Chijimatsu R. Hart D. A. Otsubo H. Shimomura K. (2017). IGF-1 Gene Transfer to Human Synovial MSCs Promotes Their Chondrogenic Differentiation Potential without Induction of the Hypertrophic Phenotype. Stem Cell Int. 2017, 1–10. 10.1155/2017/5804147 PMC5504993 28740513

[B39] Im G.-I. (2016). Endogenous Cartilage Repair by Recruitment of Stem Cells. Tissue Eng. B: Rev. 22, 160–171. 10.1089/ten.TEB.2015.0438 26559963

[B40] Jeffrey J. E. Aspden R. M. (2006). The Biophysical Effects of a Single Impact Load on Human and Bovine Articular Cartilage. Proc. Inst. Mech. Eng. H 220, 677–686. 10.1243/09544119jeim31 16961187

[B41] Jiang Y. Cai Y. Zhang W. Yin Z. Hu C. Tong T. (2016). Human Cartilage‐Derived Progenitor Cells from Committed Chondrocytes for Efficient Cartilage Repair and Regeneration. STEM CELLS Translational Med. 5, 733–744. 10.5966/sctm.2015-0192 PMC4878331 27130221

[B42] Jiang Y. Tuan R. S. (2015). Origin and Function of Cartilage Stem/progenitor Cells in Osteoarthritis. Nat. Rev. Rheumatol. 11, 206–212. 10.1038/nrrheum.2014.200 25536487 PMC5413931

[B43] Joharapurkar A. A. Pandya V. B. Patel V. J. Desai R. C. Jain M. R. (2018). Prolyl Hydroxylase Inhibitors: A Breakthrough in the Therapy of Anemia Associated with Chronic Diseases. J. Med. Chem. 61, 6964–6982. 10.1021/acs.jmedchem.7b01686 29712435

[B44] Johnson K. Zhu S. Tremblay M. S. Payette J. N. Wang J. Bouchez L. C. (2012). A Stem Cell-Based Approach to Cartilage Repair. Science 336, 717–721. 10.1126/science.1215157 22491093

[B45] Kasper G. Dankert N. Tuischer J. Hoeft M. Gaber T. Glaeser J. D. (2007). Mesenchymal Stem Cells Regulate Angiogenesis According to Their Mechanical Environment. Stem Cells 25, 903–910. 10.1634/stemcells.2006-0432 17218399

[B46] Kennedy M. I. Whitney K. Evans T. Laprade R. F. (2018). Platelet-Rich Plasma and Cartilage Repair. Curr. Rev. Musculoskelet. Med. 11, 573–582. 10.1007/s12178-018-9516-x 30203333 PMC6220001

[B47] Kim Y. G. Choi J. Kim K. (2020). Mesenchymal Stem Cell‐Derived Exosomes for Effective Cartilage Tissue Repair and Treatment of Osteoarthritis. Biotechnol. J. 15, 2000082. 10.1002/biot.202000082 32559340

[B48] Kobayashi T. Lyons K. M. Mcmahon A. P. Kronenberg H. M. (2005). BMP Signaling Stimulates Cellular Differentiation at Multiple Steps during Cartilage Development. Proc. Natl. Acad. Sci. 102, 18023–18027. 10.1073/pnas.0503617102 16322106 PMC1312369

[B49] Koh Y. G. Choi Y. J. Kwon O. R. Kim Y. S. (2014). Second-Look Arthroscopic Evaluation of Cartilage Lesions after Mesenchymal Stem Cell Implantation in Osteoarthritic Knees. Am. J. Sports Med. 42, 1628–1637. 10.1177/0363546514529641 24743139

[B50] Kwon H. Brown W. E. Lee C. A. Wang D. Paschos N. Hu J. C. (2019). Surgical and Tissue Engineering Strategies for Articular Cartilage and Meniscus Repair. Nat. Rev. Rheumatol. 15, 550–570. 10.1038/s41584-019-0255-1 31296933 PMC7192556

[B51] Lafont J. E. Talma S. Murphy C. L. (2007). Hypoxia-inducible Factor 2α Is Essential for Hypoxic Induction of the Human Articular Chondrocyte Phenotype. Arthritis Rheum. 56, 3297–3306. 10.1002/art.22878 17907154

[B52] Laternser S. Keller H. Leupin O. Rausch M. Graf-Hausner U. Rimann M. (2018). A Novel Microplate 3D Bioprinting Platform for the Engineering of Muscle and Tendon Tissues. SLAS Technol. Translating Life Sci. Innovation 23, 599–613. 10.1177/2472630318776594 PMC6249648 29895208

[B53] Lee Koh J. Kowalski A. Lautenschlager E. (2006). The Effect of Angled Osteochondral Grafting on Contact Pressure. Am. J. Sports Med. 34, 116–119. 10.1177/0363546505281236 16282582

[B54] Lee W. S. Kim H. J. Kim K. I. Kim G. B. Jin W. (2019). Intra‐Articular Injection of Autologous Adipose Tissue‐Derived Mesenchymal Stem Cells for the Treatment of Knee Osteoarthritis: A Phase IIb, Randomized, Placebo‐Controlled Clinical Trial. STEM CELLS Translational Med. 8, 504–511. 10.1002/sctm.18-0122 PMC6525553 30835956

[B55] Lefebvre V. Peeters-Joris C. Vaes G. (1990). Modulation by Interleukin 1 and Tumor Necrosis Factor α of Production of Collagenase, Tissue Inhibitor of Metalloproteinases and Collagen Types in Differentiated and Dedifferentiated Articular Chondrocytes. Biochim. Biophys. Acta (Bba) - Mol. Cel Res. 1052, 366–378. 10.1016/0167-4889(90)90145-4 2162214

[B56] Li K. Zhang C. Qiu L. Gao L. Zhang X. (2017). Advances in Application of Mechanical Stimuli in Bioreactors for Cartilage Tissue Engineering. Tissue Eng. Part B: Rev. 23, 399–411. 10.1089/ten.TEB.2016.0427 28463576

[B57] Li T. Liu B. Chen K. Lou Y. Jiang Y. Zhang D. (2020). Small Molecule Compounds Promote the Proliferation of Chondrocytes and Chondrogenic Differentiation of Stem Cells in Cartilage Tissue Engineering. Biomed. Pharmacother. 131, 110652. 10.1016/j.biopha.2020.110652 32942151

[B58] Li Z. Kupcsik L. Yao S.-J. Alini M. Stoddart M. J. (2010). Mechanical Load Modulates Chondrogenesis of Human Mesenchymal Stem Cells through the TGF-β Pathway. J. Cel Mol Med 14, 1338–1346. 10.1111/j.1582-4934.2009.00780.x PMC3828850 19432813

[B59] Liacini A. Sylvester J. Li W. Q. Huang W. Dehnade F. Ahmad M. (2003). Induction of Matrix Metalloproteinase-13 Gene Expression by TNF-α Is Mediated by MAP Kinases, AP-1, and NF-Κb Transcription Factors in Articular Chondrocytes. Exp. Cel Res. 288, 208–217. 10.1016/s0014-4827(03)00180-0 12878172

[B60] Liu M. Zeng X. Ma C. Yi H. Ali Z. Mou X. (2017). Injectable Hydrogels for Cartilage and Bone Tissue Engineering. Bone Res. 5, 17014. 10.1038/boneres.2017.14 28584674 PMC5448314

[B61] Longobardi L. O'rear L. Aakula S. Johnstone B. Shimer K. Chytil A. (2006). Effect of IGF-I in the Chondrogenesis of Bone Marrow Mesenchymal Stem Cells in the Presence or Absence of TGF-β Signaling. J. Bone Miner Res. 21, 626–636. 10.1359/jbmr.051213 16598383

[B62] Lui J. C. Colbert M. Cheung C. S. F. Ad M. Lee A. Zhu Z. (2019). Cartilage-Targeted IGF-1 Treatment to Promote Longitudinal Bone Growth. Mol. Ther. 27, 673–680. 10.1016/j.ymthe.2019.01.017 30765323 PMC6404097

[B63] Markway B. D. Cho H. Johnstone B. (2013). Hypoxia Promotes Redifferentiation and Suppresses Markers of Hypertrophy and Degeneration in Both Healthy and Osteoarthritic Chondrocytes. Arthritis Res. Ther. 15, R92. 10.1186/ar4272 23965235 PMC3979022

[B64] Mascarenhas R. Saltzman B. Fortier L. Cole B. (2015). Role of Platelet-Rich Plasma in Articular Cartilage Injury and Disease. J. Knee Surg. 28, 003–010. 10.1055/s-0034-1384672 25068847

[B65] Moldovan N. Maldovan L. Raghunath M. (2019). Of Balls, Inks and Cages: Hybrid Biofabrication of 3D Tissue Analogs. Int. J. Bioprint 5, 167. 10.18063/ijb.v5i1.167 32596531 PMC7294696

[B66] Moussa M. Lajeunesse D. Hilal G. El Atat O. Haykal G. Serhal R. (2017). Platelet Rich Plasma (PRP) Induces Chondroprotection via Increasing Autophagy, Anti-inflammatory Markers, and Decreasing Apoptosis in Human Osteoarthritic Cartilage. Exp. Cel Res. 352, 146–156. 10.1016/j.yexcr.2017.02.012 28202394

[B67] Murakami S. Lefebvre V. De Crombrugghe B. (2000). Potent Inhibition of the Master Chondrogenic FactorSox9 Gene by Interleukin-1 and Tumor Necrosis Factor-α. J. Biol. Chem. 275, 3687–3692. 10.1074/jbc.275.5.3687 10652367

[B68] Murphy C. L. Polak J. M. (2004). Control of Human Articular Chondrocyte Differentiation by Reduced Oxygen Tension. J. Cel. Physiol. 199, 451–459. 10.1002/jcp.10481 15095292

[B69] Murphy C. L. Thoms B. L. Vaghjiani R. J. Lafont J. E. (2009). HIF-mediated Articular Chondrocyte Function: Prospects for Cartilage Repair. Arthritis Res. Ther. 11, 213. 10.1186/ar2574 19232075 PMC2688225

[B70] Musumeci G. Castrogiovanni P. Leonardi R. Trovato F. M. Szychlinska M. A. Di Giunta A. (2014). New Perspectives for Articular Cartilage Repair Treatment through Tissue Engineering: A Contemporary Review. Wjo 5, 80–88. 10.5312/wjo.v5.i2.80 24829869 PMC4017310

[B71] Nawaz S. Z. Bentley G. Briggs T. W. R. Carrington R. W. J. Skinner J. A. Gallagher K. R. (2014). Autologous Chondrocyte Implantation in the Knee. J. Bone Jt. Surg Am 96, 824–830. 10.2106/jbjs.L.01695 24875023

[B72] Ngo H. X. Garneau-Tsodikova S. (2018). What Are the Drugs of the Future? Med. Chem. Commun. 9, 757–758. 10.1039/c8md90019a PMC6072476 30108965

[B73] Novo E. Valfrè di Bonzo L. Cannito S. Colombatto S. Parola M. (2009). Hepatic Myofibroblasts: a Heterogeneous Population of Multifunctional Cells in Liver Fibrogenesis. Int. J. Biochem. Cel Biol. 41, 2089–2093. 10.1016/j.biocel.2009.03.010 19782946

[B74] Okada K. Mori D. Makii Y. Nakamoto H. Murahashi Y. Yano F. (2020). Hypoxia-inducible Factor-1 Alpha Maintains Mouse Articular Cartilage through Suppression of NF-Κb Signaling. Sci. Rep. 10, 5425. 10.1038/s41598-020-62463-4 32214220 PMC7096515

[B75] Park M. S. Kim Y. H. Jung Y. Kim S. H. Park J. C. Yoon D. S. (2015). *In Situ* Recruitment of Human Bone Marrow-Derived Mesenchymal Stem Cells Using Chemokines for Articular Cartilage Regeneration. Cel Transpl. 24, 1067–1083. 10.3727/096368914x681018 24759682

[B76] Park Y.-B. Ha C.-W. Rhim J. H. Lee H.-J. (2018). Stem Cell Therapy for Articular Cartilage Repair: Review of the Entity of Cell Populations Used and the Result of the Clinical Application of Each Entity. Am. J. Sports Med. 46, 2540–2552. 10.1177/0363546517729152 29023156

[B77] Pearle A. D. Warren R. F. Rodeo S. A. (2005). Basic Science of Articular Cartilage and Osteoarthritis. Clin. Sports Med. 24, 1–12. 10.1016/j.csm.2004.08.007 15636773

[B78] Pei M. He F. (2012). Extracellular Matrix Deposited by Synovium-Derived Stem Cells Delays Replicative Senescent Chondrocyte Dedifferentiation and Enhances Redifferentiation. J. Cel. Physiol. 227, 2163–2174. 10.1002/jcp.22950 PMC3265606 21792932

[B79] Redondo M. Beer A. Yanke A. (2018). Cartilage Restoration: Microfracture and Osteochondral Autograft Transplantation. J. Knee Surg. 31, 231–238. 10.1055/s-0037-1618592 29396963

[B80] Reginato A. M. Sanz-Rodriguez C. Diaz A. Dharmavaram R. M. Jimenez S. A. (1993). Transcriptional Modulation of Cartilage-specific Collagen Gene Expression by Interferon γ and Tumour Necrosis Factor α in Cultured Human Chondrocytes. Biochem. J. 294 (Pt 3), 761–769. 10.1042/bj2940761 8379931 PMC1134527

[B81] Reissis D. Tang Q. O. Cooper N. C. Carasco C. F. Gamie Z. Mantalaris A. (2016). Current Clinical Evidence for the Use of Mesenchymal Stem Cells in Articular Cartilage Repair. Expert Opin. Biol. Ther. 16, 535–557. 10.1517/14712598.2016.1145651 26798997

[B82] Robins J. C. Akeno N. Mukherjee A. Dalal R. R. Aronow B. J. Koopman P. (2005). Hypoxia Induces Chondrocyte-specific Gene Expression in Mesenchymal Cells in Association with Transcriptional Activation of Sox9. Bone 37, 313–322. 10.1016/j.bone.2005.04.040 16023419

[B83] Sakaguchi Y. Sekiya I. Yagishita K. Muneta T. (2005). Comparison of Human Stem Cells Derived from Various Mesenchymal Tissues: Superiority of Synovium as a Cell Source. Arthritis Rheum. 52, 2521–2529. 10.1002/art.21212 16052568

[B84] Sakai N. Tager A. M. (2013). Fibrosis of Two: Epithelial Cell-Fibroblast Interactions in Pulmonary Fibrosis. Biochim. Biophys. Acta (Bba) - Mol. Basis Dis. 1832, 911–921. 10.1016/j.bbadis.2013.03.001 PMC4041487 23499992

[B85] Sakata R. Reddi A. H. (2016). Platelet-Rich Plasma Modulates Actions on Articular Cartilage Lubrication and Regeneration. Tissue Eng. Part B: Rev. 22, 408–419. 10.1089/ten.TEB.2015.0534 27109909

[B86] Salinas E. Y. Hu J. C. Athanasiou K. (2018). A Guide for Using Mechanical Stimulation to Enhance Tissue-Engineered Articular Cartilage Properties. Tissue Eng. Part B: Rev. 24, 345–358. 10.1089/ten.TEB.2018.0006 29562835 PMC6199627

[B87] Schipani E. Ryan H. E. Didrickson S. Kobayashi T. Knight M. Johnson R. S. (2001). Hypoxia in Cartilage: HIF-1α Is Essential for Chondrocyte Growth Arrest and Survival. Genes Dev. 15, 2865–2876. 10.1101/gad.934301 11691837 PMC312800

[B88] Schnabel M. Marlovits S. Eckhoff G. Fichtel I. Gotzen L. Vécsei V. (2002). Dedifferentiation-associated Changes in Morphology and Gene Expression in Primary Human Articular Chondrocytes in Cell Culture. Osteoarthritis and Cartilage 10, 62–70. 10.1053/joca.2001.0482 11795984

[B89] Sekiya I. Colter D. C. Prockop D. J. (2001). BMP-6 Enhances Chondrogenesis in a Subpopulation of Human Marrow Stromal Cells. Biochem. Biophysical Res. Commun. 284, 411–418. 10.1006/bbrc.2001.4898 11394894

[B90] Shen B. Wei A. Tao H. Diwan A. D. Ma D. D. F. (2009). BMP-2 Enhances TGF-Β3-Mediated Chondrogenic Differentiation of Human Bone Marrow Multipotent Mesenchymal Stromal Cells in Alginate Bead Culture. Tissue Eng. A 15, 1311–1320. 10.1089/ten.tea.2008.0132 18950289

[B91] Shen B. Wei A. Whittaker S. Williams L. A. Tao H. Ma D. D. F. (2009). The Role of BMP-7 in Chondrogenic and Osteogenic Differentiation of Human Bone Marrow Multipotent Mesenchymal Stromal Cells *In Vitro* . J. Cel. Biochem. 109, a–n. 10.1002/jcb.22412 19950204

[B92] Shi D. Li J. (2021). Research Progress of Fibrocartilage Hyalinization. J. Clin. Surg. 29, 388–391. 10.3969/j.issn.1005-6483.2021.04.026

[B93] Shi Y. Hu X. Cheng J. Zhang X. Zhao F. Shi W. (2019). A Small Molecule Promotes Cartilage Extracellular Matrix Generation and Inhibits Osteoarthritis Development. Nat. Commun. 10, 1914. 10.1038/s41467-019-09839-x 31015473 PMC6478911

[B94] Snoeker B. Turkiewicz A. Magnusson K. Frobell R. Yu D. Peat G. (2020). Risk of Knee Osteoarthritis after Different Types of Knee Injuries in Young Adults: a Population-Based Cohort Study. Br. J. Sports Med. 54, 725–730. 10.1136/bjsports-2019-100959 31826861

[B95] Sommerfeldt M. F. Magnussen R. A. Hewett T. E. Kaeding C. C. Flanigan D. C. (2016). Microfracture of Articular Cartilage. JBJS Rev. 4. 10.2106/jbjs.Rvw.15.00005 27486725

[B96] Sophia Fox A. J. Bedi A. Rodeo S. A. (2009). The Basic Science of Articular Cartilage: Structure, Composition, and Function. Sports Health 1, 461–468. 10.1177/1941738109350438 23015907 PMC3445147

[B97] Stöve J. Huch K. Günther K.-P. Scharf H.-P. (2000). Interleukin-1β Induces Different Gene Expression of Stromelysin, Aggrecan and Tumor-Necrosis-Factor-Stimulated Gene 6 in Human Osteoarthritic Chondrocytes *In Vitro* . Pathobiology 68, 144–149. 10.1159/000055915 11174072

[B98] Su W. Liu G. Liu X. Zhou Y. Sun Q. Zhen G. (2020). Angiogenesis Stimulated by Elevated PDGF-BB in Subchondral Bone Contributes to Osteoarthritis Development. JCI Insight 5. 10.1172/jci.insight.135446 PMC7205438 32208385

[B99] Thoms B. L. Dudek K. A. Lafont J. E. Murphy C. L. (2013). Hypoxia Promotes the Production and Inhibits the Destruction of Human Articular Cartilage. Arthritis Rheum. 65, 1302–1312. 10.1002/art.37867 23334958

[B100] Thorp H. Kim K. Kondo M. Grainger D. W. Okano T. (2020). Fabrication of Hyaline-like Cartilage Constructs Using Mesenchymal Stem Cell Sheets. Sci. Rep. 10, 20869. 10.1038/s41598-020-77842-0 33257787 PMC7705723

[B101] Toh W. S. Lai R. C. Hui J. H. P. Lim S. K. (2017). MSC Exosome as a Cell-free MSC Therapy for Cartilage Regeneration: Implications for Osteoarthritis Treatment. Semin. Cel Developmental Biol. 67, 56–64. 10.1016/j.semcdb.2016.11.008 27871993

[B102] Ulrich-Vinther M. Maloney M. D. Schwarz E. M. Rosier R. OʼKeefe R. J. (2003). Articular Cartilage Biology. J. Am. Acad. Orthopaedic Surgeons 11, 421–430. 10.5435/00124635-200311000-00006 14686827

[B103] Verteramo A. Seedhom B. B. (2007). Effect of a Single Impact Loading on the Structure and Mechanical Properties of Articular Cartilage. J. Biomech. 40, 3580–3589. 10.1016/j.jbiomech.2007.06.002 17662988

[B104] Vinod E. Vinod Francis D. Manickam Amirtham S. Sathishkumar S. Boopalan P. R. J. V. C. (2019). Allogeneic Platelet Rich Plasma Serves as a Scaffold for Articular Cartilage Derived Chondroprogenitors. Tissue and Cell 56, 107–113. 10.1016/j.tice.2018.12.006 30736898

[B105] Vonk L. A. Van Dooremalen S. F. J. Liv N. Klumperman J. Coffer P. J. Saris D. B. F. (2018). Mesenchymal Stromal/stem Cell-Derived Extracellular Vesicles Promote Human Cartilage Regeneration *In Vitro* . Theranostics 8, 906–920. 10.7150/thno.20746 29463990 PMC5817101

[B106] Wachsmuth L. Söder S. Fan Z. Finger F. Aigner T. (2006). Immunolocalization of Matrix Proteins in Different Human Cartilage Subtypes. Histol. Histopathol 21, 477–485. 10.14670/hh-21.477 16493578

[B107] Wakitani S. Imoto K. Yamamoto T. Saito M. Murata N. Yoneda M. (2002). Human Autologous Culture Expanded Bone Marrow Mesenchymal Cell Transplantation for Repair of Cartilage Defects in Osteoarthritic Knees. Osteoarthritis and Cartilage 10, 199–206. 10.1053/joca.2001.0504 11869080

[B108] Wang B. Xing D. Dong S. Tie R. Zhang Z. Lin J. (2018). Prevalence and Disease burden of Knee Osteoarthritis in China: a Systematic Review. CHINESE JOURNAL EVIDENCE-BASED MEDICINE 18, 134–142. 10.7507/1672-2531.201712031

[B109] Wang W. Rigueur D. Lyons K. M. (2014). TGFβ Signaling in Cartilage Development and Maintenance. Birth Defect Res. C 102, 37–51. 10.1002/bdrc.21058 PMC4267887 24677722

[B110] Wang Y. Sun X. Lv J. Zeng L. Wei X. Wei L. (2017). Stromal Cell-Derived Factor-1 Accelerates Cartilage Defect Repairing by Recruiting Bone Marrow Mesenchymal Stem Cells and Promoting Chondrogenic Differentiation. Tissue Eng. Part A 23, 1160–1168. 10.1089/ten.TEA.2017.0046 28478702 PMC6037190

[B111] Wehling N. Palmer G. D. Pilapil C. Liu F. Wells J. W. Müller P. E. (2009). Interleukin-1β and Tumor Necrosis Factor α Inhibit Chondrogenesis by Human Mesenchymal Stem Cells through NF-κb-dependent Pathways. Arthritis Rheum. 60, 801–812. 10.1002/art.24352 19248089 PMC2688727

[B112] Weiss C. Rosenberg L. Helfet A. J. (1968). An Ultrastructural Study of normal Young Adult Human Articular Cartilage. J. Bone Jt. Surg. 50, 663–674. 10.2106/00004623-196850040-00002 5658553

[B113] Welch T. Mandelbaum B. Tom M. (2016). Autologous Chondrocyte Implantation: Past, Present, and Future. Sports Med. Arthrosc. Rev. 24, 85–91. 10.1097/jsa.0000000000000115 27135292

[B114] Wilder F. V. Hall B. J. Barrett J. P. Jr. Lemrow N. B. (2002). History of Acute Knee Injury and Osteoarthritis of the Knee: a Prospective Epidemiological Assessment. Osteoarthritis and Cartilage 10, 611–616. 10.1053/joca.2002.0795 12479382

[B115] Wu M. Hu R. Wang J. An Y. Lu L. Long C. (2019). Salidroside Suppresses IL-1β-Induced Apoptosis in Chondrocytes via Phosphatidylinositol 3-Kinases (PI3K)/Akt Signaling Inhibition. Med. Sci. Monit. 25, 5833–5840. 10.12659/msm.917851 31381554 PMC6691749

[B116] Wu W. Zhang J. Dong Q. Liu Y. Mao T. Chen F. (2009). Platelet-rich Plasma - A Promising Cell Carrier for Micro-invasive Articular Cartilage Repair. Med. Hypotheses 72, 455–457. 10.1016/j.mehy.2008.11.032 19138823

[B117] Xu X. Shi D. Shen Y. Xu Z. Dai J. Chen D. (2015). Full-thickness Cartilage Defects Are Repaired via a Microfracture Technique and Intraarticular Injection of the Small-Molecule Compound Kartogenin. Arthritis Res. Ther. 17, 20. 10.1186/s13075-015-0537-1 25641548 PMC4376363

[B118] Yano F. Hojo H. Ohba S. Fukai A. Hosaka Y. Ikeda T. (2013). A Novel Disease-Modifying Osteoarthritis Drug Candidate Targeting Runx1. Ann. Rheum. Dis. 72, 748–753. 10.1136/annrheumdis-2012-201745 23041841

[B119] Yuan L. Sakamoto N. Song G. Sato M. (2013). Low-level Shear Stress Induces Human Mesenchymal Stem Cell Migration through the SDF-1/CXCR4 axis via MAPK Signaling Pathways. Stem Cell Development 22, 2384–2393. 10.1089/scd.2012.0717 23544621

[B120] Zhai G. Doré J. Rahman P. (2015). TGF-β Signal Transduction Pathways and Osteoarthritis. Rheumatol. Int. 35, 1283–1292. 10.1007/s00296-015-3251-z 25773660

[B121] Zhang R. Ma J. Han J. Zhang W. Ma J. (2019). Mesenchymal Stem Cell Related Therapies for Cartilage Lesions and Osteoarthritis. Am. J. Transl Res. 11, 6275–6289. 31737182 PMC6834499

[B122] Zhang Y. Zhang J. Chang F. Xu W. Ding J. (2018). Repair of Full-Thickness Articular Cartilage Defect Using Stem Cell-Encapsulated Thermogel. Mater. Sci. Eng. C 88, 79–87. 10.1016/j.msec.2018.02.028 29636141

[B123] Zhang Y. Zhao Q. (2018). Salidroside Attenuates Interleukin‐1β‐induced Inflammation in Human Osteoarthritis Chondrocytes. J. Cel Biochem 120, 1203–1209. 10.1002/jcb.27076 30270563

[B124] Zhao Z. Li Y. Wang M. Zhao S. Zhao Z. Fang J. (2020). Mechanotransduction Pathways in the Regulation of Cartilage Chondrocyte Homoeostasis. J. Cel Mol Med 24, 5408–5419. 10.1111/jcmm.15204 PMC7214151 32237113

[B125] Zhen G. Guo Q. Li Y. Wu C. Zhu S. Wang R. (2021). Mechanical Stress Determines the Configuration of TGFβ Activation in Articular Cartilage. Nat. Commun. 12, 1706. 10.1038/s41467-021-21948-0 33731712 PMC7969741

[B126] Zhen G. Wen C. Jia X. Li Y. Crane J. L. Mears S. C. (2013). Inhibition of TGF-β Signaling in Mesenchymal Stem Cells of Subchondral Bone Attenuates Osteoarthritis. Nat. Med. 19, 704–712. 10.1038/nm.3143 23685840 PMC3676689

[B127] Zhou C. Zheng H. Seol D. Yu Y. Martin J. A. (2014). Gene Expression Profiles Reveal that Chondrogenic Progenitor Cells and Synovial Cells Are Closely Related. J. Orthop. Res. 32, 981–988. 10.1002/jor.22641 24797716 PMC6415308

[B128] Zhou Y. Tao H. Li Y. Deng M. He B. Xia S. (2016). Berberine Promotes Proliferation of Sodium Nitroprusside-Stimulated Rat Chondrocytes and Osteoarthritic Rat Cartilage via Wnt/β-Catenin Pathway. Eur. J. Pharmacol. 789, 109–118. 10.1016/j.ejphar.2016.07.027 27445236

